# Supplementary material for: GABAB receptor auxiliary subunits modulate Cav2.3-mediated release from medial habenula terminals
Source: eLife. 2021 Apr 29;10:e68274. doi: 10.7554/eLife.68274 (PMC8121548; doi:10.7554/eLife.68274)
Supplement: Supplementary file 1. — Gmax is the maximal conductance density. *Gmax was significantly increased in KCTD8-transfected cells compared with Control (p=0.0340, one-way ANOVA with Tukey post hoc test); Vrev is the reversal potential; V0.5 act is the potential at which current density was half-maximal; Kα is the slope factor. [file elife-68274-supp1.docx]

**Supplementary File 1 for:**

**GABA_B_ receptor auxiliary subunits modulate Cav2.3-mediated release from medial habenula terminals**

Authors: Pradeep Bhandari^1^, David Vandael^1^, Diego Fernandez-Fernandez^2^, Thorsten Fritzius^2^, David Kleindienst^1^, Cihan Önal^1^, Jacqueline Montanaro^1^, Martin Gassmann^2^, Peter Jonas^1^, Akos Kulik^3,4^, Bernhard Bettler^2^, Ryuichi Shigemoto^1^*, Peter Koppensteiner^1^*

Affiliations: ^1^Institute of Science and Technology (IST) Austria, 3400 Klosterneuburg, Austria ^2^Department of Biomedicine, University of Basel, 4056 Basel, Switzerland ^3^Institute of Physiology II, Faculty of Medicine, ^4^BIOSS Centre for Biological Signalling Studies, University of Freiburg, 79104 Freiburg, Germany

*Corresponding Authors: [ryuichi.shigemoto@ist.ac.at](mailto:ryuichi.shigemoto@ist.ac.at) and [peter.koppensteiner@ist.ac.at](mailto:peter.koppensteiner@ist.ac.at)

|  | **Control (n = 14)** | **KCTD8 (n = 10)** | **KCTD12b (n = 8)** |
| --- | --- | --- | --- |
| **G_max_ (nS/pF)** | 0.75 ± 0.10 | 1.17 ± 0.12 * | 0.91 ± 0.15 |
| **V_rev_ (mV)** | 63.48 ± 3.08 | 66.5 ± 3.00 | 60.55 ± 3.84 |
| **V_0.5 act_ (mV)** | -2.54 ± 2.28 | -3.93 ± 1.64 | -2.46 ± 1.78 |
| **K_α_** | 8.01 ± 1.33 | 7.15 ± 1.03 | 7.05 ± 1.77 |

**Supplementary Table 1:**

Parameters of the Boltzmann fit shown in Figure 8C. G_max_ is the maximal conductance density. * G_max_ was significantly increased in KCTD8-transfected cells compared with Control (P = 0.0340, one-way ANOVA with Tukey post hoc test); V_rev_ is the reversal potential; V_0.5 act_ is the potential at which current density was half-maximal; K_α_ is the slope factor
